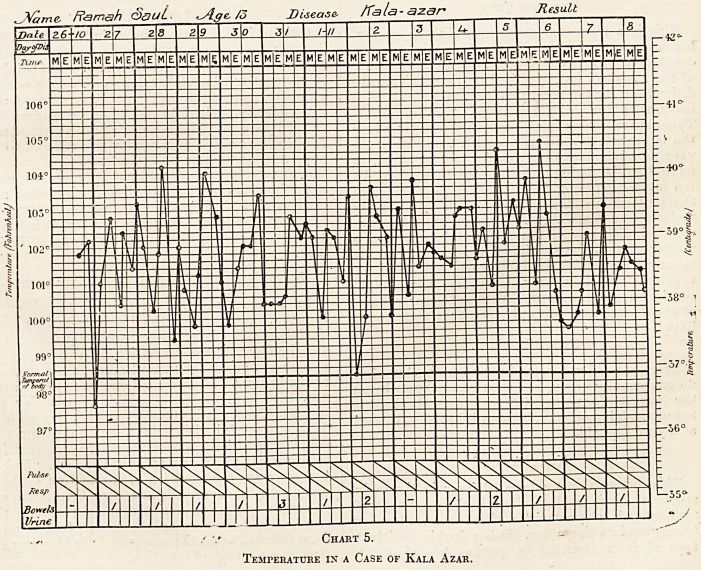# The Clinical Diagnosis of Fevers in the Tropics

**Published:** 1907-07-27

**Authors:** Leonard Rogers

**Affiliations:** Professor of Pathology, Calcutta


### HOSPITAL CLINICS.

### THE CLINICAL DIAGNOSIS OF FEVERS IN THE TROPICS.

Clinical Lecture at the Post Graduate College.

By MAJOR LEONARD ROGERS, M.D., F.R.C.P., F.R.C.S., I.M.S., Professor of Pathology, Calcutta.

THE lecturer said that during the last few years he examined carefully many fever cases in different parts of India; and, from this comprehensive inquiry he obtained a series of temperature charts and curves of the disease, which led him to the conclusion that it is possible to diagnose nearly all fevers by clinical methods. Clinically, fevers are divided into short and long ones, of which the great majority are of the former type.

CHART 1.
SHOWING TEMPERATURE IN A CASE OF BENIGN TERTIAN.

### MALARIAL FEVERS.

*Quartan Malaria.*—Typical charts of single quartan malarial fever show pyrexia every third day, but as a matter of fact the typical chart is never seen except in untreated cases or text-books Quinine causes the temperature to fall, and it rarely rises again. In two years he met with but one typical chart, that of a patient admitted to hospital for phthisis.

### BENIGN TERTIAN.

The characteristic rise of temperature occurs every second day in the case of a single infection, and the pyrexia lasts from 12 to 16 or 20 hours, leaving a considerable interval before the next rise, which is of similar duration; in this way it differs from malignant tertian. The fever is entirely modified by the administration of quinine. The great majority of the cases are double infections (see chart 1), in which the temperature rises every day on account of two groups of parasites being present, and the two infections may show themselves separately on the chart. When quinine is administered the pyrexia never lasts for more than two or three days. It disappears in from eight to sixteen or twenty hours. In benign tertian cases fully a fourth only get a single paroxysm.

CHART 2.
TEMPERATURE IN A CASE OF MALIGNANT TERTIAN.

### MALIGNANT TERTIAN.

In malignant tertian the temperature rises rapidly and remains up for over 24 hours, intermits for a very short time, and then goes up again, and this happens every second day (see chart 2). There is a little depression on the height of the curve, which is a common characteristic of the malignant tertian curve.

CHART 3.
TEMPERATURE IN A CASE OF SEVEN DAY FEVER.

### MALARIA.

The pulse is rapid in cases of malaria—110 to 140. The temperature does not often run over 104° F. As a matter of fact, if malignant tertians are treated with quinine, more than one of the typical rises is seldom seen. The quinine is given in 10-grain doses by the mouth three to six times a day. Sometimes this form of malaria simulates the quotidian type. One of the charts shows a regular remittent type of fever resembling typhoid, but, when the blood was examined, numerous parasites were found. This is a dangerous fever, and unless it is treated promptly the patient may die within three or four hours, although he may not at first show any very serious symptoms. These cases ought to be recognised at the earliest possible period, and quinine administered intravenously. In ordinary cases 30 or 60 grains by the mouth acts perfectly efficiently. A ten dose of quinine, when the number of parasites in the blood is large, is rapid and effective in its action when given intravenously.

### DURATION OF MALIGNANT FEVERS.

There ought to be no such thing as malignant malarial fever lasting four or six days under quinine. If the fever lasts longer, something else should be

CHART 4.
TEMPERATURE IN A CASE OF TYPHOID FEVER.

suspected. Malignant tertian always soon succumbs to quinine, nor does it ever approach a continued type of fever. A chronic malarial fever is one which has been untreated by quinine. The maximum rise of malarial cases in Calcutta is at the end of the rains, during the drying-up period.

### The Seven-Day Fever.

This fever closely simulates Dengue. At a discussion in Calcutta on this subject recently, the practitioners who had seen dengue unanimously regarded this disease as being totally distinct from seven-day fever. It has been prevalent in Calcutta for a number of years, and has been confused with malaria and with simple continued fever. In over 90 per cent. of the cases it lasts six or seven days, begins with a rigor, which it is very exceptional to find repeated. With the onset of the fever malaise of influenzal type occurs, but not the pains or swollen joints described in epidemic dengue. The chart shows a saddleback kind of reaction, and nearly always a marked terminal rise (see chart 3). Sponging has no permanent effect on the temperature. The pulse is slow, 76 to 90, even when the temperature is between 103° and 104°. This is very characteristic of the seven-day fever, and is not the case in true malaria. The pyrexia will continue its course whatever treatment is adopted. Another important feature is that the fever may be typhoid in type for a time, and the two conditions further resemble one another in the slow pulse rate. I can find in no description of dengue anything like this saddle-back kind of chart. A very large number of cases come to hospital showing only the later stages of the disease, and the chart does not then indicate the initial rise, but an apparent decline with the terminal rise. In these cases slow pulse (84-92) with a temperature of 103-105°, will aid the diagnosis. Sometimes the terminal rise only is seen. In simple cases of this fever no malarial parasites are found in the blood.

### Epidemic Dengue.

Charts of a true epidemic dengue, taken in 1872, show how the fever falls rapidly on the second day and generally remains normal. The pulse in this disease is invariably rapid, 110 to 140, or more. In dengue you always get breakbone pains going on for weeks and months afterwards; the patient is lucky if he gets rid of the effects of the disease in two or three months. In seven-day fever no such after-pains are present.

Chart 5.

Temperature in a Case of Kala Azar.

### Influenza.

In influenza one meets with pulmonary and other symptoms which are not present in the seven-day fever, nor is temperature of the saddle-back type. A heatstroke chart shows a temperature rising up to 109, the patient recovering with the ordinary cold-bath treatment, but the temperature continued intermittent for some days afterwards, due, no doubt, to the shock to the heat centres. There is an absolute causal relationship between the temperature and moisture conditions and the incidence of heatstroke, and no statement to the contrary made by writers as to the microbic character of these diseases can stand examination.

### Relapsing Fever.

This is common in Bombay, and occurs occasionally in the Punjab. The temperature comes down with a crisis and remains subnormal for two or three days; after an interval of about a week it rises again, and there may be a third or even a fourth rise. The pulse-rate varies with the temperature. When the blood is examined, spirilla are found. This is one of the long fevers, and in it there is marked leucocytosis. This does not as a rule occur in malaria or in Malta fever, with which relapsing fever may be confused.

### Typhoid Fever.

This is a most important fever in the tropics, because so many other fevers found in tropical countries confuse the diagnosis, but a large number of the cases can be recognised by the clinical conditions and the temperature chart. One important feature is the high continued type of fever. Typhoid fever is a typical continued fever in which the temperature remains over 101°, and does not vary more than two degrees for two or three, or more days, becoming normal by lysis (see chart 4). An interesting point in this case is the absence of the large mono-nuclear increase which occurs in malaria and Kala Azar. Typhoid may be complicated with any of the malarial fevers. As a matter of fact the continued type seldom occurs in any fever which is liable to be mistaken for typhoid. In Kala Azar, for example, the high-continued type is never met with in the early stages of the disease, and it is only then that confusion is likely.

### Short Typhoid.

These are difficult cases in the tropics, but they do occasionally occur, and it is necessary to be on the look-out for them. Clinically, paratyphoid is not of much importance, because it has to be treated as a pure typhoid.

### Kala Azar.

This disease shows a type of fever in the early stages which is liable to be mistaken for that of typhoid. A characteristic chart shows two or three rises in a single day. It is a double remittent type of fever resistant to quinine (see chart 5). It can be differentiated from typhoid by certain points. The fever may be of a low continued type very rare in typhoid. The blood examination shows nothing very definite, but cultures can be made from the venous blood in broth. In about one month the spleen commences to enlarge, often extending to the navel, and then there is no doubt about the type of disease. Late in the disease there may be high continued fever, accompanied by sloughing of the nose or mouth, and death at the end of six months. This disease affords a terrible example of the impotence of medicine. After long periods, even months, of a low continued fever a double remittent type may come on again. In some cases patients lose the fever for a time, and are even permanently cured by an intercurrent septic malady. The high continued type is very dangerous, and cancrum oris frequently occurs. One case of recovery which took place after a septic infection led to the hope that it would be possible to cure this disease by the injection of sterilised cultures of staphylococcus, according to the method of Sir A. E. Wright. In all the cases so treated marked increase of leucocytes resulted. In one case in which the leucocytes were less than 500 per cubic millimetre, and 5 per cent. were polynuclear, within three weeks they increased nearly to the normal number after an attack of cancrum oris and the patient recovered.

### Quinine in Kala Azar.

Quinine is of the utmost value in this disease, but it must be given over long periods in doses of not less than 50 to 90 grains a day. One child of eight years, to which 50 grains a day were given for six weeks, increased from 32 to 46 lb. in 46 days, and got perfectly well. The mortality in Madras has been 98 per cent.; in Assam, where this method was used in 500 cases, the mortality was reduced to 75 per cent. As we have no better method of treating this disease, quinine should not be neglected, even if the dose has to be guarded with strychnine and cardiac tonics. The disease attacks children in enormous numbers. We now know that it is due to a special organism discovered by Professor Leishman.

### Some Further Points in its Treatment.

In chronic cases, cirrhosis of the liver is common, and sometimes the disease assumes a dropsical type; the later stages are frequently characterised by extreme emaciation. The germs of the disease have been found by Patten in the stomach of the bedbug. Bedbugs fed on cases of kala azar, are, therefore, probably capable of carrying the disease. Hence it is necessary to disinfect all beds with a powerful disinfectant, and in Assam this simple precaution has been the means of preventing the disease in previously infected houses. As long as ten years ago the adoption of segregation methods was a complete success in stamping the disease out of certain tea gardens in Assam. As some of the segregation huts were only from 200 to 400 yards from infected areas the mosquito is in this way excluded as a carrier of infection.

## Figures and Tables

**Chart 1. f1:**
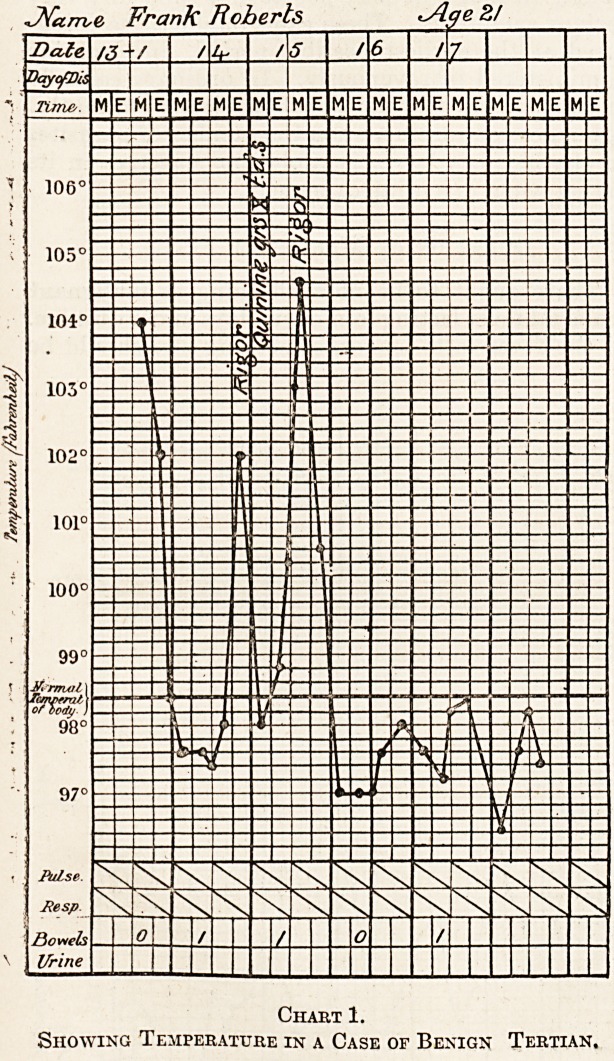


**Chart 2. f2:**
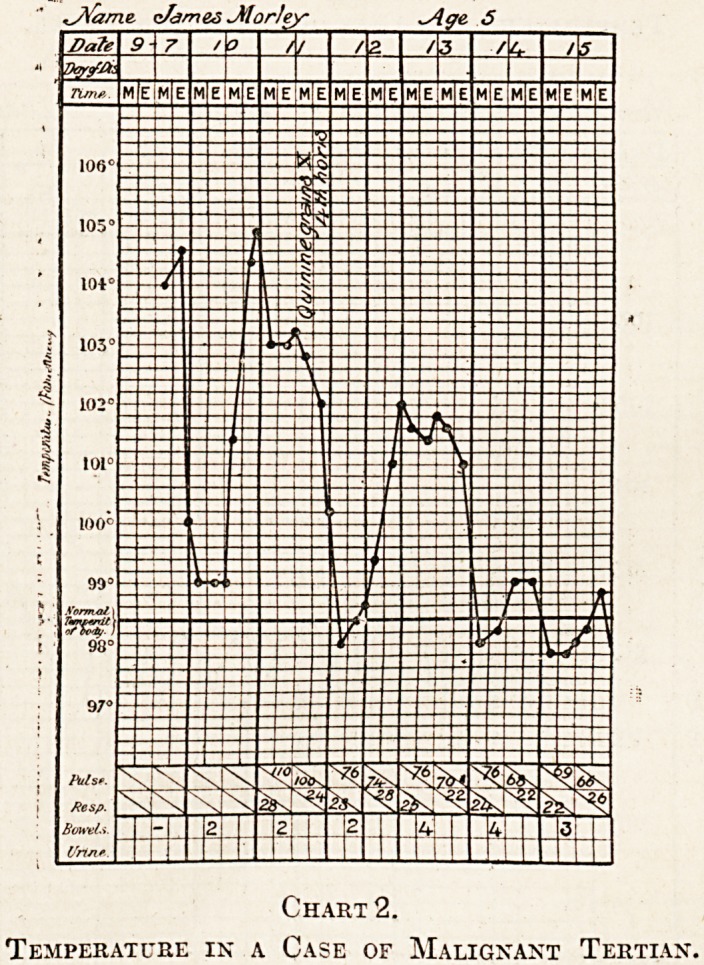


**Chart 3. f3:**
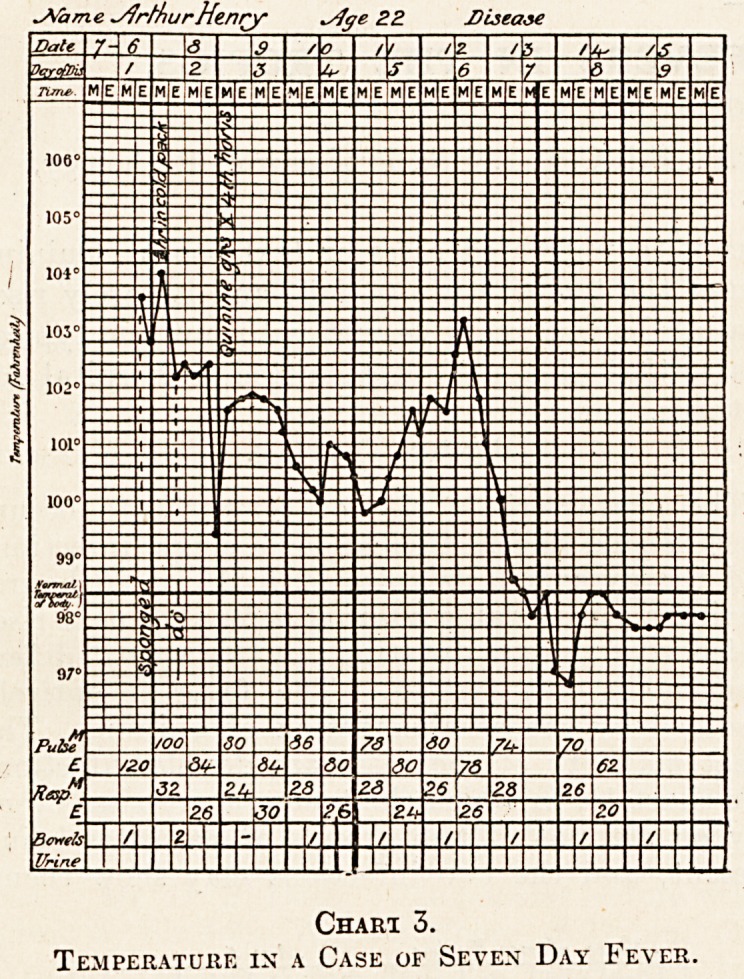


**Chart 4. f4:**
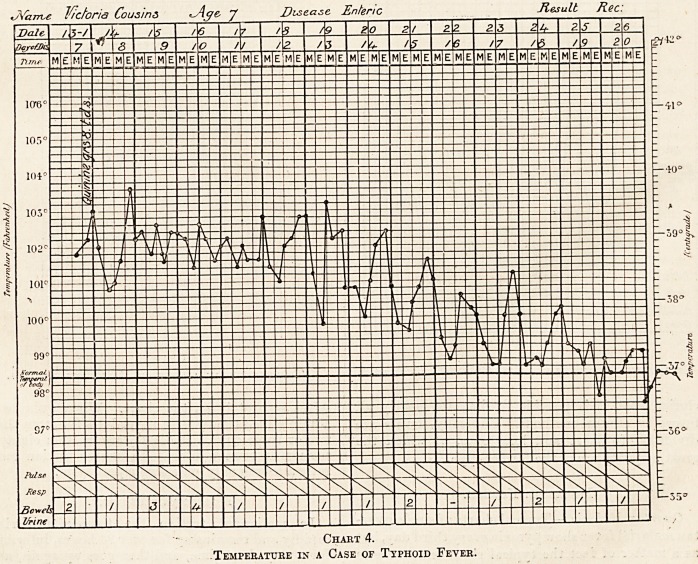


**Chart 5. f5:**